# Automated Evaluation of Upper Airway Obstruction Based on Deep Learning

**DOI:** 10.1155/2023/8231425

**Published:** 2023-02-18

**Authors:** Yunho Jeong, Yeeyeewin Nang, Zhihe Zhao

**Affiliations:** State Key Laboratory of Oral Diseases & National Clinical Research Center for Oral Diseases, Department of Orthodontics, West China Hospital of Stomatology, Sichuan University, Chengdu, Sichuan 610041, China

## Abstract

**Objectives:**

This study is aimed at developing a screening tool that could evaluate the upper airway obstruction on lateral cephalograms based on deep learning.

**Methods:**

We developed a novel and practical convolutional neural network model to automatically evaluate upper airway obstruction based on ResNet backbone using the lateral cephalogram. A total of 1219 X-ray images were collected for model training and testing.

**Results:**

In comparison with VGG16, our model showed a better performance with sensitivity of 0.86, specificity of 0.89, PPV of 0.90, NPV of 0.85, and F1-score of 0.88, respectively. The heat maps of cephalograms showed a deeper understanding of features learned by deep learning model.

**Conclusion:**

This study demonstrated that deep learning could learn effective features from cephalograms and automated evaluate upper airway obstruction according to X-ray images. *Clinical Relevance*. A novel and practical deep convolutional neural network model has been established to relieve dentists' workload of screening and improve accuracy in upper airway obstruction.

## 1. Introduction

Upper airway obstruction can result in reduction of breathing or impediment of gas exchange, and it is usually associated with sleep-disordered breathing (SDB) [1, 2]. The cause of upper airway obstruction includes polyps, environmental irritants, allergic rhinitis, and adenotonsillar hypertrophy [3, 4]. Increasing evidence has shown an association between dentofacial anomalies and obstruction in upper airway. Lopatiene et al. analyzed examination results including dental casts and radiographs of 49 children with respiratory obstruction, and they found significant link between nasal resistance and increase overjet, open bite, and maxillary crowding [5]. Children with upper airway obstruction may manifest as mouth breathing, which can result in narrow maxilla, mandibular skeletal retrognathism, increased lower facial height, and high palate [6]. Most children with this type of malocclusion and craniofacial deformity present to dental clinics complaining of occlusal disorder or dissatisfaction with their profile. Lateral cephalogram was a useful and common tool for dentists to evaluate the severity of upper airway obstruction. Although there are multiple other tools applied to assess upper airway obstruction, including computed tomography (CT), fluoroscopy, magnetic resonance imaging (MRI), and fibreoptic pharyngoscopy; lateral cephalometry is still an appealing approach for screening upper airway obstruction in dental clinics as it is a cheap and easily available technique with less radiation and certain diagnostic value [7–9]. Cephalometric analysis based on McNamara method is a classical measurement for airway analysis [10]. However, the landmark-label process is time- and energy-consuming even for a senior orthodontist. Besides, this experience-dependent technique is difficult to master for young dentists and dentists who have not investigated cephalometric measurements such as endodontists or prosthodontists, which may lead to missed or delayed diagnosis. Thus, it would be useful for orthodontists to develop an automated evaluation method to improve efficiency in upper airway obstruction using lateral cephalograms.

In the past several years, automated methods based on deep learning have achieved excellent results in diagnosis, segmentation, detection tasks, and so on [11–13]. For example, Mahdi et al. proposed a residual network-based faster R-CNN model to recognize teeth and evaluate both positional relationship and confidence score of the candidates. This model showed an F1-score of 0.982 which indicated that deep learning was useful and reliable for dental assistance [14]. Similarly, deep learning was also used for identifying the brand and model of a dental implant from a radiograph with a sensitivity of 93.5% and a specificity of 94.2% [15]. In orthodontics, accurate skeletal classification can assist orthodontists in making treatment plans. Yu et al. developed a deep learning model for skeletal classification solely from the lateral cephalogram. In that work, deep learning learned from X-ray imaging labeled by human experts and exhibited superior performance with >90% sensitivity, specificity, and accuracy for vertical and sagittal skeletal diagnosis [16].

In this paper, we developed a novel deep convolutional neural network (DCNN) based on ResNet backbone for automated evaluation of upper airway obstruction. Here, our study was novel with 4 main contributions. Firstly, this is the first research to evaluate upper airway obstruction based on deep learning using lateral cephalograms with high sensitivity and specificity. Secondly, the heat maps of cephalograms showed a deeper understanding of features learned by deep learning model. This visualization provided interpretable information in upper airway obstruction based on deep learning. Finally, our model is lite and practical and can be deployed in fundamental clinics with less memory and computational overhead.

## 2. Materials and Methods

### 2.1. Dataset

**Figure 1:**
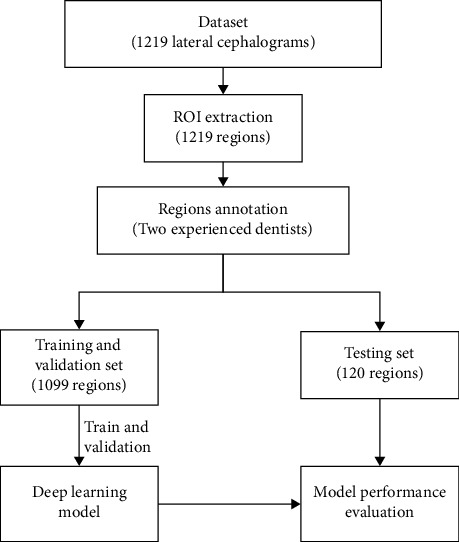
Study flowchart.

A study flowchart of our study was presented in Figure 1. Cephalometric radiographs were retrospectively examined for 1783 cohorts who had initially visited our hospital between March and September 2019. We first excluded the low-quality images (324 images) and eliminated images (240 images) where the anatomic structure (soft palate, tongue, or pharyngeal wall) was difficult to recognize. Finally, a total of 1219 X-ray images of cohorts with lateral X-ray examination were obtained, of which the numbers of upper airway obstruction and nonobstruction were 610 and 609, respectively. Cephalometric radiographs were taken from X-ray machine Morita ×550 (Tube energy 80 kV, Tube current 10 mA; Morita, Kyoto, Japan). The distance between X-ray plate and X-ray machine was 180 cm, and the resolution of X-ray images is 1752 × 1537 (22.9 cm × 20.1 cm). We cropped the airway region from the center to the bottom of original X-ray images with a resolution of 1000 × 500. The airway regions of X-ray images were randomly divided into 2 groups: a training set (1099 images) and a testing set (120 images). Demographic data are shown in Table 1.

**Figure 2:**
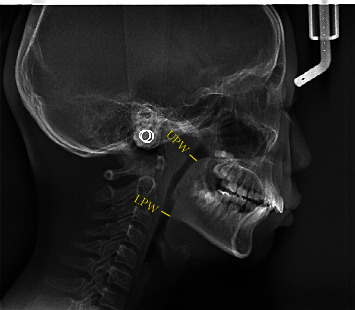
Illustration of cephalometric measurements for upper airway. Upper pharyngeal width (UPW): linear distance from a point on the posterior wall of the soft palate (the anterior half part) to the posterior pharyngeal wall where there was the greatest closure of the airway. Lower pharyngeal width (LPW): measured from an intersection point of the posterior border of the tongue and the lower border of mandible to the closest point on the posterior pharyngeal wall.

The McNamara method was considered a classic cephalometric analysis for evaluation of upper airway dimension [10]. Linear measurements were performed using Image J software (Rasband software, W.S., Image J, National Institutes of Health, Bethesda, MD, http://rsb.info.nih.gov/ij/). Many studies have applied McNamara method for assessment of upper airway, which can be divided into nasopharynx (upper pharynx) and oropharynx (lower pharynx) [17–19]. The upper pharyngeal width (UPW) was measured linearly from a point on the posterior wall of the soft palate to the posterior pharyngeal wall where there was the greatest closure of the airway. The measurement of the lower pharyngeal width (LPW) was a linear distance from an intersection point of the posterior border of the tongue and the lower border of mandible to the closest point on the posterior pharyngeal wall [17] (Figure 2). In our research, X-ray images of patients with mixed dentition were manually labelled as “obstruction” (UPW <12 mm or LPW <10 mm) or “nonobstruction” (UPW ≥ 12 mm and LPW ≥ 10 mm), and the images of patients with permanent dentition were manually labelled as “obstruction” (UPW <17.4 mm or LPW <10 mm) or “nonobstruction” (UPW ≥ 17.4 mm and LPW ≥ 10 mm). All the measurements were carried out by the two experienced dentists blinded by each other. A third senior orthodontic specialist with 30 years of experience was consulted in cases of disagreement. If the three experts still could not get an agreement, the confusing image would be excluded.

### 2.2. Deep Learning Model

**Figure 3:**
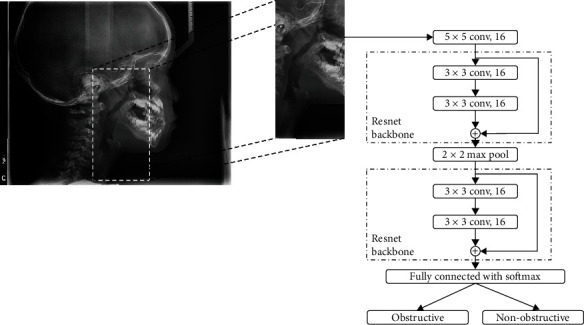
Preprocessing and model architecture.

ResNet-18 is a famous model and achieved excellent results in the field of image classification [20]. In this model, the backbone structure consists of two convolutional layers with skip connection. This backbone has been proven that it has a strong capacity of feature extraction in the image classification task. Hence, we chose ResNet-18 as the backbone to develop a lite and practical model for automated evaluation of upper airway obstruction. Regarding our model, the kernel size of two convolutional layers is 3×3 and its stride is 1. To overcome overparameterization problem, we not only reduce the size and the number of kernels but also reduce the number of backbone blocks. The kernel number is 16 in the first backbone and the second backbone. Max pooling with the size of 2 × 2 is deployed between two backbone blocks. The features of airway region are extracted in the first extra convolutional layer with 5 × 5 kernel and 1 stride. And the feature maps produced by first layer are given to backbone structure. At the end of backbone structure, fully-connected layer with softmax switched feature maps into the probability of obstruction or nonobstruction. The rectified linear unit (ReLU) is included in every convolutional layer. The architecture of model is shown in Figure 3.

### 2.3. Statistical Analysis and Evaluation Criteria

To better measure the performance of the model, we used evaluation metrics of sensitivity (SEN), specificity (SPEC), positive predictive value (PPV), negative predictive value (NPV) and F1-score [21–24]. And the metrics equations are calculated as follows:

(1) $$\begin{array}{c} \text{SEN}=\frac{\text{TP}}{\text{TP}+\text{FN}},\\ \text{SPEC}=\frac{\text{TN}}{\text{FP}+\text{TN}},\\ \text{PPV}=\frac{\text{TP}}{\text{TP}+\text{FP}},\\ \text{NPV}=\frac{\text{TN}}{\text{FN}+\text{TN}},\\ \text{F}1\text{ score}=\frac{2*\text{PPV}*\text{SEN}}{\text{PPV}+\text{SEN}}, \end{array}$$

where the TP, FP, TN, and FN indicated true positive, false negative, true negative, and false negative, respectively. Positive/negative means that the model predicts that the X-ray image is obstructive/nonobstructive, and true/false means that the prediction is right/wrong. In these metrics, the F1-score is the most overall metric which indicates the harmonic mean of PPV and SEN. The highest possible value of F1-score is 1, indicating perfect PPV and SEN, and the lowest possible value is 0, if either PPV or SEN is zero. All value of metrics is ranged from 0 to 1.

For deeper understanding of the feature in X-ray images, the heat map with class activation mapping (CAM) was generated according to the method proposed by Zhou et al. [25]. This map visually highlights the cephalogram region that is most informative in evaluation of upper airway obstruction.

## 3. Results

The study was developed using 1099 X-ray images for training and 120 X-ray images for testing. All experiments were performed in Python 3.6 and TensorFlow 1.9 on a single NVIDIA RTX 2080Ti [26]. We randomly selected 100 images from training set as a validation set to observe training situation and obtain the highest performance. In the training phase, we used a learning rate of 0.001 in the Adam optimizer and used the “Cross-Entropy” loss function with the batch size of 50. After 30 epochs, automatic evaluation of upper airway obstruction was performed using the testing dataset. In many dental applications, VGG16 is a popular model for classification and diagnosis [27, 28]. Hence, we also carried out experimental comparison between VGG16 and DCNN. In the testing set, DCNN model showed 0.86 sensitivity, 0.89 specificity, 0.90 PPV, 0.85 NPV, and 0.88 F1-score, respectively. DCNN showed higher performance than VGG16 in our study (Table 2).

**Figure 4:**
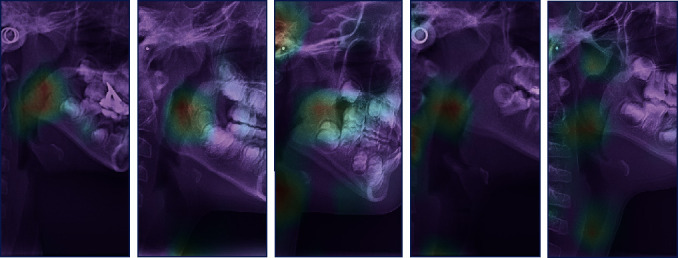
Class activation maps. The red area shows key features discovered by deep learning.

Figure 4 shows the heat maps created with class activation mapping. This is an indication of a well-trained model that effectively uses the information in the cephalogram. According to the heat maps, the upper airway area was activated when model received a sample with airway obstruction. This activated area revealed that model taught itself according to human annotated conclusion without extra orientation. The processing speed of DCNN was about 5 s for analyzing 120 lateral cephalograms with a single NVIDIA RTX 2080Ti graphic processing unit.

## 4. Discussion

Upper airway obstruction, as a hot topic studied by dentists and otolaryngologists, showed an intimate association with malocclusion and development of craniofacial complex, and it was also the main etiological factor of obstructive sleep apnea syndrome (OSAS) in children [29]. OSAS may lead to problems which were harmful to children, such as inattention, poor learning, failure to thrive, or even pulmonary hypertension [30]. However, missed or delayed diagnosis was common as signs and symptoms of children were not clear and the experience-dependent diagnosis method was difficult to master [31]. For children who were in an early stage of mental and physical development, airway patency and sleep quality were significant. So, developing a timely and accurate screen system for upper airway obstruction was advantageous.

As a two-dimensional analysis method, lateral cephalometric images have long been discussed about its reliability in assessing pharyngeal volumes. A study consisting of 36 prepubertal children ranging from 4.9–9.8 years old compared the validity of upper airway using MRI and cephalometric measurements, and researchers found that cephalometric measurements showed significant correlations with MRI measurements. The authors concluded that the cephalometric radiograph was a useful screening tool when evaluating nasopharyngeal or retropalatal airway size [9]. Besides, cephalometric analysis was also investigated as a useful tool to evaluate OSAS patients [32]. In this paper, we developed a novel and practical DCNN model to automatically evaluate upper airway obstruction using the lateral cephalogram. Our data demonstrated that deep learning method was able to evaluate upper airway obstruction with high accuracy and improve screening efficiency in dental clinics.

To the best of our knowledge, so far, there is only one research that is similar to our research, which applied artificial intelligence technology to detect patients with severe obstructive sleep apnea based on cephalometric radiographs [33]. However, it only focused on the oropharynx but not on the nasopharynx. Some research reported automatic segmentation of the airway space with convolutional neural network on CBCT images [34, 35]. We must admit that CBCT offers information on cross-sectional areas, volume, and 3D form that cannot be determined by cephalometric images. However, many studies have confirmed the screening value of cephalometric images [9, 32], which possesses lots of advantages, including lower cost, and less radiation dose. Besides, cephalometric images are more wildly used by dentists, especially in developing countries and clinics which cannot afford CBCT machines.

In our study, a DCNN model based on ResNet backbone was applied for automated evaluation of upper airway obstruction. Our experimental results revealed that a simplified model can overcome overparameterization problems to some extent. In dental applications, the size of dental image dataset is always smaller than natural image datasets generally, since the acquisition of data requires the authorization of the patient. Additionally, professional knowledge is required in the work of data annotation. Thus, it is difficult to collect enough high-quality dental samples for training and testing. Under this condition, a typical model like VGG16 with a large number of parameters is suffered from insufficient data so that it can easily overfit the dataset. To avoid the defects mentioned above, we used ResNet as the backbone to construct a simplified DCNN model, which showed better performance than VGG16.

Although labeling landmarks in the lateral cephalogram were important for orthodontic diagnosis [36–38], errors in landmark identification method are widespread, necessitating time-consuming manual correction. Hence, we applied deep learning method to directly classify X-ray images rather than identification key point methods. Our model showed good performance in both sensitivity and specificity. Moreover, our model did not require extra steps of feature extraction for training or prediction. The heat maps (Figure 4) also confirmed that the well-trained model can discover abnormal area in X-ray images by itself without extra processing.

Nevertheless, our study presented several limitations. First, although 1219 was a large number within the realm of dental research, it was far less than the application requirement of deep learning. Second, our 1219 X-ray images were produced by Morita ×550 at one resolution. Our model may not be robust at other resolutions, which should be addressed through appropriate expansion of the training set with images at other resolutions. Last but not the least, major improvements for the sensitivity and specificity of our research may be achieved in the future by increasing sample size, applying advanced architectures, optimal training strategies, and data generation.

## 5. Conclusions

This study presents a deep learning model that can automatically detect upper airway obstruction with higher accuracy and more time-efficiency, which would reduce the burden on dentists in clinical work. A simplified DCNN model based on ResNet backbone structure showed good performance for automatic evaluation of upper airway obstruction based on the lateral cephalogram. However, deep learning is not completely accurate in the detection of upper airway obstruction. To avoid false negative diagnosis, regular follow-ups and reevaluations are required if necessary.

## Figures and Tables

**Table 1:** Clinical and demographic characteristics of study cohorts.

Characteristic	Training set (*N* = 1099)	Testing set (*N* = 120)
Median age (range)	12.15 (7-18)	12.02 (7-18)
Sex		
Male	545	54
Female	554	66
Clinical evaluation		
Obstructive	550	60
Nonobstructive	549	60

**Table 2:** The performance of deep learning model.

Operator	SEN	SPEC	PPV	NPV	F1-score
VGG16	0.84	0.86	0.86	0.84	0.85
ResNet-18	0.83	0.86	0.87	0.82	0.85
EfficientNet	0.84	0.88	0.88	0.83	0.86
MobileNet v2	0.85	0.88	0.88	0.85	0.87
DCNN (ours)	0.86	0.89	0.90	0.85	0.88

## Data Availability

The datasets generated and/or analyzed during the current study are not publicly available due to data security but are available from the corresponding author on reasonable request.
